# Views of people with disordered eating on current and alternative out-of-home calorie labelling policies in England: a mixed-methods survey

**DOI:** 10.1136/bmjph-2025-003666

**Published:** 2026-04-15

**Authors:** Nora Trompeter, Abinaya Nadarajah, Helen Sharpe, Fiona Duffy, Ellen Maloney, Dasha Nicholls, Lucy Serpell, Ulrike Schmidt, Tom Jewell

**Affiliations:** 1 Institute of Child Health, University College London, London, UK; 2 Florence Nightingale Faculty of Nursing Midwifery and Palliative Care, King’s College London, London, UK; 3 Department of Clinical Psychology, School of Health in Social Sciences, University of Edinburgh, Edinburgh, Scotland; 4 NHS Lothian Child and Adolescent Mental Health Services, Royal Edinburgh Hospital, Edinburgh, Scotland; 5 Department of Brain Sciences, Imperial College London, London, UK; 6 Department of Clinical, Educational and Health Psychology, University College London, London, UK; 7 North East London NHS Foundation Trust, Rainham, UK; 8 Centre for Eating and Weight Disorders, King's College London, London, UK; 9 Eating Disorders Outpatients Service, South London and Maudsley NHS Foundation Trust, London, UK; 10 Psychological and Mental Health Services, Great Ormond Street Hospital NHS Foundation Trust, London, UK

**Keywords:** Public Health, Obesity, Mental Health

## Abstract

**Introduction:**

Out-of-home calorie labelling was introduced in England as part of public health policy to address obesity. We examined the acceptability and perceived impacts of the policy on individuals with disordered eating. Additionally, potential alternative nutritional label policies were explored. We hypothesised that individuals with binge eating would view the policy more favourably compared with individuals without binge eating.

**Methods:**

A cross-sectional mixed-methods study was conducted from February 2024 to March 2025. Inclusion criteria were age ≥16 years, living in England and having experienced disordered eating. In total, 1001 people participated.

**Results:**

Latent profile analyses revealed five distinct groups of people based on policy acceptability, perceived impacts and avoidance of calorie labels: (1) highly negative with avoidance (n=202, 20.2%); (2) highly negative without avoidance (n=112, 11.2%); (3) moderately negative (n=183, 18.3%); (4) neutral (n=241, 24.1%) and (5) positive (n=263, 26.3%). Current binge eating was associated with lower likelihood of belonging in the *highly negative with avoidance* (OR 0.45 (0.30; 0.69)) or *highly negative without avoidance* groups (OR 0.45 (0.26; 0.78)) and a higher likelihood of belonging in the *neutral* (OR 1.84 (1.26; 2.69)) or *positive* groups (OR 2.04 (1.42; 2.93)). Thematic analysis generated four superordinate themes: *reassurance*, *emotional distress*, *criticisms* and *impacts on recovery*. When ranking different nutritional label policies, optional calorie labels were the most popular policy, with 63.6% ranking the policy in their top three options.

**Conclusions:**

People with disordered eating hold diverse opinions on out-of-home calorie labels. Our findings shed new light on which demographic groups are negatively or positively impacted by such labels and suggest that optional calories on menus would be the preferred policy choice of people with disordered eating.

**WHAT IS ALREADY KNOWN ON THIS TOPIC**

- Mandatory calorie and/or nutrition labelling for out-of-home food consumption has been implemented in several countries.
- Individuals with lived experience of disordered eating have reported primarily negative impacts to date.

**WHAT THIS STUDY ADDS**

- This study generated a more comprehensive and nuanced understanding of the impact of calorie labelling on individuals with lived experience of disordered eating.
- The study also investigates preferences for different types of nutritional labels.

**HOW THIS STUDY MIGHT AFFECT RESEARCH, PRACTICE OR POLICY**

- Findings can inform policy decisions on whether out-of-home calorie labelling policies strike the balance between potential benefits and harms at population level.
- This study provides evidence to policy makers on the acceptability of alternative nutrition labelling policies from the perspective of people with disordered eating.

##  Introduction

In response to rising obesity rates, England introduced a public health policy in 2022 requiring calorie labels in out-of-home food outlets (eg, restaurants). Specifically, the policy applies to large businesses with ≥250 employees and states that calorie labelling in kilocalories and reference to the recommended daily intake (ie, 2000 kcal) is required on menus and other points of choice.^1^ Similar policies have previously been introduced in the USA, Canada and Australia.^2^ Recent evidence suggests that, while these types of policies lead to small reductions in calorie intake in the general population,^3^ negative mental health impacts have been observed among people with eating disorders.^4^,^6^ With rising rates of eating disorders and high associated costs to the health system,^7 8^ potential benefits to public health through obesity reduction may therefore be outweighed by potential harms to people with eating disorders. The current study sought to examine heterogeneity in the acceptability and perceived impacts of the calorie label policy, as well as views on alternative out-of-home labelling policies, among people with lived experience of eating disorders and disordered eating. By disordered eating, we refer to the broad spectrum of eating disorder symptoms, including those meeting criteria for a diagnosable eating disorder, as well as sub-threshold presentations.

Previous studies examining the impact of England’s out-of-home calorie label policy have found that while most people with eating disorders report negative impacts, some benefits have also been observed.^4^ For instance, some people with eating disorders have reported that out-of-home calorie labels can reinforce eating disorder symptoms^5 9^ and contribute to weight stigma,^6^ while others have reported feeling reassured by seeing calorie information.^6 9^ Moreover, studies in the USA have shown that individuals with restrictive eating both pay more attention to calorie labels on menus compared with individuals who do not engage in restrictive eating and are more likely to choose lower calorie options when provided with calorie labels, effects not seen among people without restrictive eating.^10 11^ However, no study has been able to examine this heterogeneity to determine whether certain groups of individuals are at greater or lesser risk of negative consequences. Additionally, existing research has focused on singular aspects relating to either the acceptability of the policy or the impact on mental health and behaviours. While such a variable-centred approach is useful in providing initial insights, it does not account for the relationship between those aspects. A person-centred approach examining acceptability and impacts simultaneously to construct specific impact profiles among individuals with disordered eating may be more informative for public policy and clinical practice.^12^

Thus, the current study aimed to examine the acceptability and perceived impacts of England’s calorie label policy on individuals with disordered eating and to explore potential alternative nutritional label policies for the out-of-home sector. Based on prior research,^4^ we hypothesised that individuals with binge eating would view the policy more favourably compared with individuals without binge eating. All other analyses were designed to be hypothesis-generating. Findings from the study aim to provide critical evidence informing inclusive public health strategies that minimise harm while supporting informed food choices, both within England and the international context.

## Methods

### Participants and recruitment

Participants were recruited through three distinct sources to ensure diverse experiences were included: (1) social media and professional networks, (2) research database (ie, Eating Disorder Genetics Initiative (EDGI) study)^13^ and (3) a paid research platform (ie, Prolific Academic).^14^ We aimed to recruit 200–300 participants per recruitment source. Participants were eligible if they were aged ≥16 years, currently lived in England and had ever experienced eating disorder symptoms, including at a subclinical level. The study was advertised as seeking views from people who had experiences with eating disorder symptoms. In particular, as treatment-seeking rates for eating disorders are low^15 16^ and help-seeking is more likely among women, people with lower body mass index and those with restrictive eating disorders,^17^,^19^ we did not require a formal diagnosis of an eating disorder for participation to increase the representativeness of the sample. Efforts were made to present the study as seeking diverse views on the out-of-home calorie label policy to minimise participation bias. The policy has previously received criticisms on social media^20^ and has been campaigned against by England’s leading eating disorder charity.^21^ All study materials were developed in collaboration with a lived experience advisory group.

In total, 1001 participants completed the current study (social media: n=375, EDGI: n=327 and Prolific: n=299). See online supplemental figure S1 for further information on recruitment. However, we were unable to calculate the response rate as we do not know how many unique eligible individuals received or viewed the study invitation.

### Measures

#### Sociodemographic information

We collected information on the following sociodemographic characteristics: age, gender identity, ethnicity, geographic location, occupation, education level and self-reported height (in cm or feet/inches) and weight (in kg or stones). BMI in kg/m^2^ was used to categorise participants as underweight (<18.5), normal weight (18.5–24.9), overweight (25–29.9) and obese (≥30).

#### Clinical characteristics

Participants were asked a range of questions to ascertain their history of eating disorders, comorbid conditions and current functioning. These included self-identified diagnosis of eating disorders and comorbid disorders, formal eating disorder diagnosis, history of eating disorder treatment, self-identification of neurodivergence and current impairment (as measured by the Clinical Impairment Assessment Questionnaire (CIA) social and personal subscales).^22^

#### Eating disorder symptoms

All participants reported on their current eating disorder symptoms using the Eating Disorder Diagnostic Scale (EDDS). The scale is a 22-item self-report measure designed to generate probable diagnoses of anorexia nervosa, bulimia nervosa and binge eating disorder.^23^ Items cover attitudinal symptoms (eg, fear of fatness), binge eating behaviours (eg, loss of control) and compensatory behaviours (eg, fasting). For the current study, we used both the overall diagnostic scale and single items capturing specific eating disorder symptoms (eg, binge eating). Operationalisation of Diagnostic and Statistical Manual of Mental Disorders, fifth edition, eating disorder diagnoses from EDDS responses is shown in the online supplemental table S1. Given the low prevalence of binge eating disorder in the sample, this was combined with probable binge eating disorder for analysis in the current study.^24 25^

#### Acceptability and impact of calorie policy

Participants were asked a range of questions regarding the calorie label policy. The questions explored participants’ overall views on calories on menus, the impact the policy has had on them, reasons for seeking out or avoiding calorie-labelled outlets and how these changes have affected them, if at all. Questions were developed in collaboration with both a lived experience panel and public health stakeholders. Questions were presented as Likert-style options on a five-point scale ranging from *strongly support*/*very positive* to *strongly oppose*/*very negative*. Free-text response questions allowed participants to elaborate on their responses.

#### Ranking of policy options

Participants were asked to rank eight different nutritional labelling policies in order of preference, whereby all options were ranked, and no ties were allowed. These included calorie labels, calorie labels as optional (eg, use a QR code to view calorie information), traffic light labels, physical exercise equivalents, Nutri-Score labels, health star ratings, low calorie stickers and healthy choice ticks. An example sheet was provided with visual examples of all options. A free-text response question allowed participants to elaborate on their response.

### Procedure

The study protocol and analysis were preregistered (https://osf.io/54s8c/?view_only=a967cc8bbdff4470b231b3a4c64556fe), and all deviations are clearly outlined below. A copy of the full questionnaire is also available on the OSF site. The study was open from February 2024 to March 2025. Participants from social media and EDGI were able to self-select into the study based on a short screening survey and were offered the opportunity to enter a draw for one of five £50 gift cards. For Prolific participants, the study was open to participants who currently lived in England and reported a previous diagnosis of an eating disorder. Prolific participants were reimbursed in line with platform guidelines (£10 per participant). All responses were collected through the online platform Qualtrics.

To reduce the likelihood of bot responses, social media recruitment was implemented as a two-step approach whereby only the link for a screening survey was shared, and participants required a valid email address to receive the actual survey link. All participants had to complete Google reCAPTCHA to screen out bot responses (score <0.5).^26^ Open-text responses were screened for suspected artificial intelligence (AI)-generated text. Copy/paste responses were disallowed on Qualtrics for the Prolific participants. The issue of AI-generated text responses was discussed only in the lead-up to the Prolific study launch. Hence, we employed extra precautions in the survey for Prolific participants but did not change our procedure for the other recruitment channels.

### Patient and public involvement

Individuals with lived experience of eating disorders were involved at multiple stages of this research. They contributed to the initial design of the study by helping to ensure that the study’s aims reflected priorities important to the eating disorder community. They reviewed and provided feedback on participant materials, including recruitment information sheets and questionnaire wording, to ensure that language and content were accessible and sensitive. They also reviewed drafts of the manuscript and helped shape the reporting and dissemination plans to make the findings more relevant and useful to those affected by eating disorders, their families and practitioners.

### Data analysis

First, we report overall descriptive information for the sample before examining latent groups representing different views on the calorie label policy using latent class analysis with the following indicator variables: perceptions of labels, impacts of policy and change in behaviours. We tested models with 2–5 groups, as further complexity was not supported by the data. While we had planned to use an ordinal link function, the models did not converge, and the data were treated continuously instead. Prior to analysing the three indicator variables, they were centred around 0. All analyses were conducted in Stata V.18.

The best fit for the different models was determined using the following criteria^27^: (1) Bayesian Information Criterion (BIC) with lower values indicating better model fit, (2) Akaike Information Criterion (AIC) with lower values indicating better model fit, (3) entropy value with higher values indicating better classification quality and (4) theoretically meaningful interpretation. Once classes were established, group differences were tested using multinomial regression analyses. While we originally planned to use analysis of variance (ANOVA), a regression framework was deemed more appropriate for use with covariates. In addition to the planned analyses, exploratory analyses were conducted to determine if any demographic or clinical characteristics were uniquely associated with group membership. For the analyses, all variables were included simultaneously to determine potential unique associations. All analyses controlled for participant source (ie, social media, EDGI or Prolific) and participation date. Levels of missing data were low across all variables of interest (<2%).

Open-ended responses to four survey questions were analysed using reflexive thematic analysis. An inductive approach was taken, allowing themes to be developed from the content of participants’ responses. Themes were developed iteratively through close reading and interpretative coding. AN and TJ independently generated initial themes and reviewed these collaboratively. Codes were reviewed together with NT, who acted as a ‘critical friend’ in the process.^28^ All authors are familiar with research on calorie labelling and eating disorders and brought shared reflexive awareness to the analysis process. The research team consists of people with clinical, academic and lived experience of eating disorders. Our overall position is that the out-of-home calorie labelling policy likely has diverse impacts on people with eating disorders.

Lastly, we examined ranked policy options by investigating popularity using the mean rank, median rank and marginal rank and controversy using the range and SD.

## Results

### Sample characteristics

Participants were predominantly female, reported themselves as white and had a degree-level qualification (see table 1). Most participants had received an eating disorder diagnosis from a health professional (n=652, 65.2%), see table 1 for details. Overall, more participants opposed the policy (50% strongly or somewhat oppose) compared with supporting it (27% strongly or somewhat support), and more participants reported negative impacts (62% very or somewhat negative) compared with reporting positive impacts (19% very or somewhat positive). However, fewer people reported specifically avoiding food outlets with calorie labels (ie, larger businesses). See online supplemental figure S2 for further details.

**Table 1:** Sample characteristics

	N/M	Percentage/SD
Gender		
Man	67	6.69%
Trans man	15	1.50%
Woman	873	87.21%
Non-binary	41	4.10%
Ethnicity		
Asian	77	7.69%
Black	21	2.10%
Mixed	50	5.00%
White	835	83.42%
Other	12	1.20%
Age in years	31.99	(12.17)
Education		
GCSEs or equivalent	47	4.70%
AS, A levels or equivalent	214	21.38%
NVQ levels 1–3 or equivalent	57	5.69%
Degree level or above	674	67.33%
Employment		
Employed full-time	443	44.26%
Employed part-time	201	20.08%
Not working due to full-time education	202	20.18%
Not working due to caretaking responsibilities	16	1.60%
Unemployed	63	6.29%
Other	70	6.99%
Weight status		
Underweight	146	14.59%
Normal weight	423	42.26%
Overweight	107	10.69%
Obese	325	32.47%
Body mass index	25.45	(9.16)
Recovery status		
Fully recovered	178	18.45%
Partially recovered	589	61.04%
Not recovered	198	20.52%
CIA social	6.74	(4.60)
CIA personal	12.21	(5.13)
Probable diagnosis		
No eating disorder/low risk	166	16.58%
Anorexia nervosa	119	11.89%
Bulimia nervosa	104	10.39%
Binge eating disorder	74	7.39%
OSFED—binge eating/purging type	259	25.87%
OSFED—low frequency binge eating disorder	13	1.30%
OSFED—restricting type	266	26.57%
Purging frequency		
Not at all	733	73.23%
1–2 times per week	121	12.09%
More than twice a week	147	14.69%
Binge eating frequency		
Not at all	419	41.86%
1–2 times per week	319	31.87%
More than twice a week	263	26.27%
Excessive exercise frequency		
Not at all	584	58.40%
1–2 times per week	183	18.30%
More than twice a week	233	23.30%
Fasting frequency		
Not at all	450	45.05%
	215	21.52%
More than twice a week	334	33.43%
Weight/shape concerns	5.03	(1.47)
Current comorbid disorder		
No	348	34.77%
Yes	653	65.23%
Past comorbid disorder		
No	551	55.04%
Yes	450	44.96%
Neurodivergent		
Yes	473	48.51%
No	502	51.49%

AS, Advanced Subsidiary; CIA, Clinical Impairment Assessment Questionnaire ; GCSE, General Certificate of Secondary Education; NVQ, National Vocational Qualification; OSFED, other specified feeding or eating disorder.

In terms of helpfulness of calorie labels, 37% of people rated them helpful in fast-food outlets, 34.6% in coffee shops and 39.1% in sit-down restaurants. In contrast, 49% of people rated labels unhelpful in fast-food outlets, 55.9% in coffee shops and 62.9% in sit-down restaurants.

### Latent class analysis

**Figure 1:**
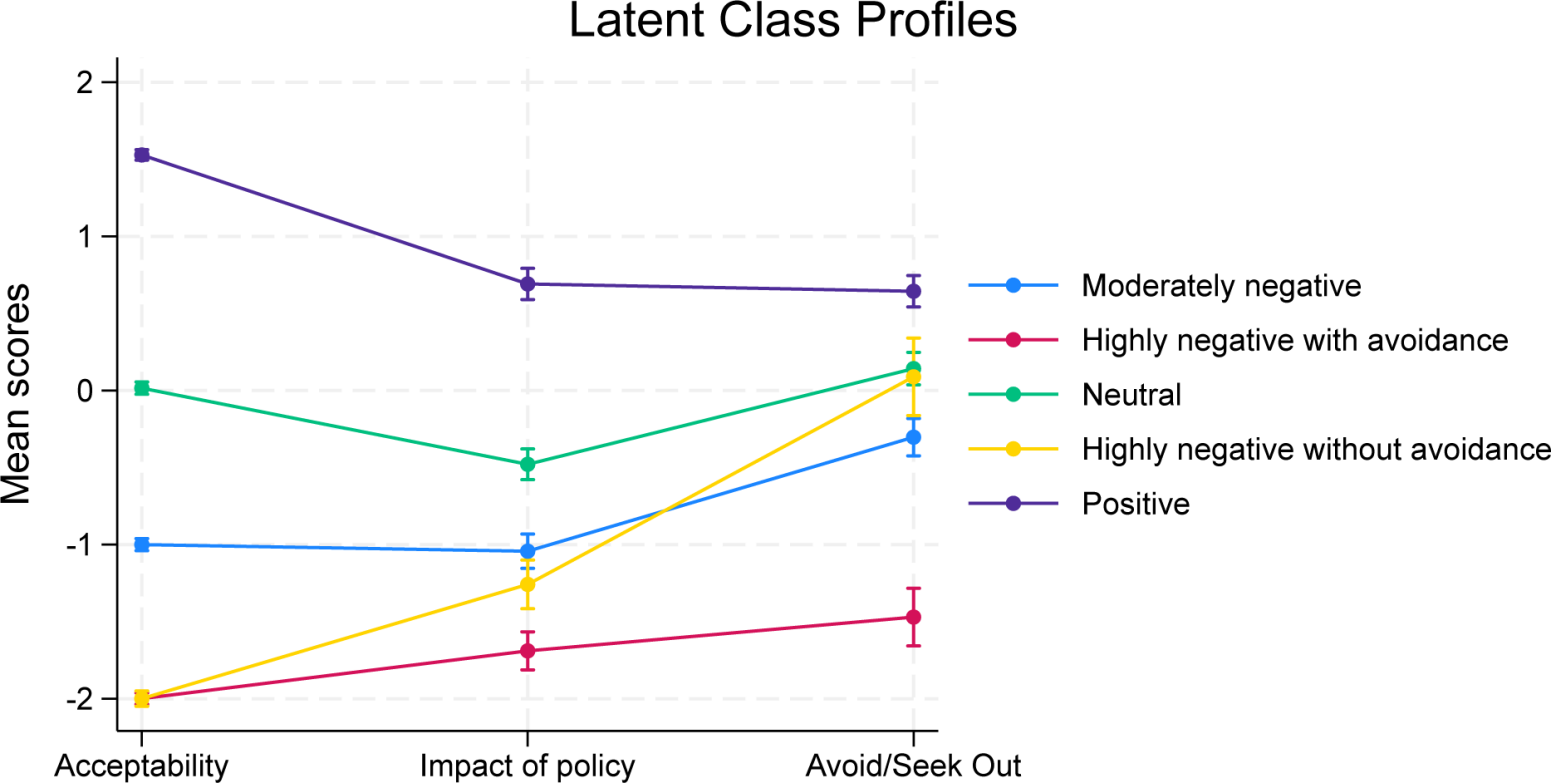
Latent profile plot based on indicator variables.

The latent class analysis model with five groups showed the best fit to the data and demonstrated interpretable differences between the groups (see figure 1 and online supplemental table S2). Specifically, the groups were labelled as (1) highly negative with avoidance (n=202, 20.2%); (2) highly negative without avoidance (n=112, 11.2%); (3) moderately negative (n=183, 18.3%); (4) neutral (n=241, 24.1%); and (5) positive (n=263, 26.3), whereby *avoidance* refers to avoiding food outlets which have calorie labels on their menus. Demographic characteristics of the groups are presented in online supplemental table S3.

Membership of the *highly negative with avoidance* group was more likely for people with non-binary gender, lower BMI, neurodivergence, higher clinical impairment, having received treatment for an eating disorder, not currently engaging in binge eating and meeting criteria for other specified feeding or eating disorder (OSFED) restrictive type (see table 2). Membership of the *highly negative without avoidance* group was more likely for women, people with non-binary gender, those in full recovery compared with not being recovered, those with lower clinical impairment, those not currently engaging in binge eating and experiencing low weight/shape concerns. Membership of the *moderately negative* group was more likely for people with lower clinical impairment in social activities. Membership of the *neutral* group was more likely for people who had not received treatment for an eating disorder and were currently engaged in binge eating. Membership of the *positive* group was more likely for older people, men, people with higher BMIs, those who had not received treatment for an eating disorder, those currently engaged in binge eating more than twice a week and those who had higher levels of weight/shape concerns.

**Table 2:** Odds ratios comparing probability of group membership of latent classes compared with any other group.

	Highly negative with avoidance versus other	Highly negative without avoidance versus other	Moderately negative versus other	Neutral vs other	Positive versus other
Age in years	0.98 (0.97; 1.00)	0.99 (0.97; 1.00)	0.98 (0.97; 1.00)	1.00 (0.99; 1.02)	**1.03 (1.02; 1.04)**
Gender					
Women	2.31 (1.04; 5.16)	**8.71 (1.19; 63.59)**	1.10 (0.61; 2.00)	0.89 (0.53; 1.49)	**0.47 (0.29; 0.76)**
Non-binary	**4.71 (1.73; 12.87)**	**10.19 (1.18; 88.36)**	1.26 (0.50; 3.20)	0.44 (0.16; 1.18)	**0.31 (0.13; 0.76)**
Body mass index	**0.95 (0.93; 0.98**)	0.99 (0.96; 1.02)	0.99 (0.96; 1.01)	1.00 (0.99; 1.02)	**1.04 (1.02; 1.05)**
Neurodivergent (yes)	**1.70 (1.22; 2.35**)	0.86 (0.57; 1.31)	0.69 (0.49; 0.97)	0.85 (0.62; 1.16)	1.08 (0.80; 1.45)
Current comorbid disorder	1.40 (0.99; 1.98)	0.61 (0.40; 0.92)	1.10 (0.77; 1.58)	1.15 (0.83; 1.58)	0.81 (0.60; 1.11)
Past comorbid disorder	1.19 (0.87; 1.63)	0.77 (0.52; 1.16)	1.19 (0.86; 1.65)	0.95 (0.71; 1.27)	0.90 (0.68; 1.20)
Recovery status					
Partially recovered	1.59 (1.01; 2.50)	0.59 (0.37; 0.95)	1.09 (0.69; 1.71)	0.83 (0.56; 1.23)	1.09 (0.73; 1.63)
Not recovered	1.78 (1.04; 3.10)	**0.28 (0.13; 0.59)**	1.11 (0.64; 1.91)	0.78 (0.48; 1.28)	1.33 (0.83; 2.15)
Social impairment	**1.07 (1.04; 1.11)**	**0.93 (0.89; 0.97)**	**0.95 (0.91; 0.99)**	1.00 (0.97; 1.03)	1.02 (0.99; 1.06)
Personal impairment	**1.04 (1.01; 1.08)**	**0.94 (0.90; 0.97)**	0.96 (0.93; 1.00)	1.00 (0.97; 1.03)	1.03 (1.00; 1.06)
Treatment received	**3.11 (2.13; 4.53)**	1.69 (1.09; 2.63)	0.84 (0.60; 1.18)	**0.67 (0.49; 0.90)**	**0.57 (0.42; 0.77)**
Binge eating					
Once or twice	**0.47 (0.32; 0.69)**	0.55 (0.34; 0.88)	1.00 (0.69; 1.46)	**1.92 (1.34; 2.73)**	1.50 (1.06; 2.13)
More than twice	**0.45 (0.30; 0.69)**	**0.45 (0.26; 0.78)**	0.76 (0.50; 1.16)	**1.84 (1.26; 2.69)**	**2.04 (1.42; 2.93)**
Fasting					
Once or twice	1.06 (0.69; 1.62)	0.54 (0.31; 0.96)	0.78 (0.51; 1.20)	1.18 (0.81; 1.72)	1.33 (0.92; 1.92)
More than twice	1.53 (1.07; 2.20)	0.73 (0.46; 1.17)	0.64 (0.44; 0.94)	1.04 (0.74; 1.47)	1.13 (0.81; 1.57)
Purging					
Once or twice	1.06 (0.65; 1.75)	0.90 (0.47; 1.71)	0.85 (0.51; 1.42)	0.92 (0.58; 1.46)	1.22 (0.80; 1.87)
More than twice	1.36 (0.89; 2.09)	0.63 (0.32; 1.21)	0.80 (0.50; 1.30)	1.01 (0.67; 1.53)	1.10 (0.74; 1.64)
Excessive exercise					
Once or twice	1.39 (0.91; 2.11)	0.58 (0.31; 1.08)	0.92 (0.59; 1.41)	1.00 (0.68; 1.47)	1.03 (0.71; 1.51)
More than twice	1.61 (1.11; 2.34)	1.07 (0.66; 1.71)	0.78 (0.52; 1.17)	0.77 (0.53; 1.12)	1.00 (0.70; 1.41)
Weight/shape concerns	1.01 (0.91; 1.13)	**0.73 (0.66; 0.82)**	0.97(0.67; 1.78)	1.05 (0.95; 1.17)	**1.25 (1.11; 1.41)**
Probable anorexia nervosa	1.60 (1.03; 2.48)	0.86 (0.45; 1.62)	1.10 (0.67; 1.78)	1.08 (0.69; 1.68)	0.58 (0.35; 0.94)
Probable bulimia nervosa	0.45 (0.24; 0.87)	0.47 (0.20; 1.10)	1.29 (0.79; 2.13)	1.11 (0.69; 1.77)	1.59 (1.03; 2.46)
Probable binge eating disorder	0.45 (0.22; 0.92)	0.99 (0.48; 2.05)	0.88 (0.49; 1.58)	0.93 (0.55; 1.57)	**1.85 (1.17; 2.93)**
Probable OSFED restrictive type	**1.67 (1.20; 2.33)**	1.13 (0.73; 1.74)	0.70 (0.48; 1.04)	0.86 (0.61; 1.21)	0.88 (0.63; 1.22)
Probable OSFED binge/purge type	0.68 (0.46; 1.00)	0.73 (0.44; 1.22)	0.85 (0.58; 1.25)	1.26 (0.91; 1.75)	1.36 (0.99; 1.87)

Notes. ORs <1 indicate lower likelihood of being in the respective category compared with any other category. ORs >1 indicate greater likelihood of being in the respective category compared any other category. Significant associations are bolded at p<0.01. All analyses controlled for recruitment source and participation date.

OSFED, other specified feeding or eating disorder.

Exploratory analyses looking at unique associations showed that for membership of the *negative with avoidance* group, only history of eating disorder treatment (OR 2.23 (1.41; 3.54)) remained significant, whereby having received treatment for an eating disorder was associated with a higher likelihood of being in the *negative with avoidance* group compared with any other group. For membership in the *negative without avoidance* group, only low weight/shape concerns (OR 0.56 (0.43; 0.72)) remained significant, whereas meeting criteria for OSFED restrictive type was significantly associated with a higher likelihood of group membership (OR 4.56 (1.68; 12.36)) despite not being significant in univariate analyses. For membership of the *moderately negative* group, younger age (OR 0.97 (0.95; 0.99)) and not being recovered (OR 2.96 (1.35; 6.45)) remained significantly associated with group membership. For membership of the *neutral* group, currently engaging in binge eating once or twice a week (OR 2.19 (1.34; 3.59)) and more than twice a week (OR 2.40 (1.39; 4.16)) remained significantly associated with group membership. In contrast, for the *positive* group, only older age (OR 1.03 (1.01; 1.04)) and being a man as opposed to a woman (OR 2.35 (1.36; 4.05)) remained significant.

### Qualitative analyses

Qualitative findings highlighted both potential benefits and harm of calorie labels (see table 3). There were four key themes identified: reassurance, emotional distress, criticism and impact on recovery*. Reassurance* was an overarching theme in which people reported appreciating the informed choice that calorie labels provided, feeling safe from the knowledge about calorie content, providing them a sense of control and facilitating them to eat out socially. In contrast, *emotional distress* captured people’s anxiety and guilt around food choices, feeling judged by others and negative impacts on social experiences. Several people also reported *criticism*, citing concerns with calorie labels as indicators of nutritional content, voicing their frustration with the policy decision and questioning the balance of the general population’s needs with the needs of people with eating disorders. *Impact on recovery* captured people being triggered by calorie information, finding the labels facilitated their eating disorder thoughts and behaviours, fear around relapse, challenges to their recovery and ambivalence on the perceived helpfulness or unhelpfulness of calorie labels at different stages of their recovery.

**Table 3:** Thematic structure

Themes	Subthemes	Illustrative quote/s
Reassurance	Make an informed choice	‘Calories on menus have actually helped me not go over my calorie intake for the day and helped me make better eating choices. I find it very informative and wish calories were listed sooner’. (P535)
	Safety through knowledge	‘I feel safer knowing how many calories are in something—anxiety is reduced when I don't have to estimate’.(P962) ‘Helped me understand that food calories isn't a negative thing. Sometimes the foods I love are not as high in calories as I assume. It’s helped me be better with my eating and put things in context’. (P494)
	A sense of control	‘It gives me control knowing how many I have consumed and do I can be accurate about my daily intake’. (P1084) ‘Having calorie information on a menu makes me feel safe and able to make choices and to work around them in my day to day life so I have no extreme reactions and relapse into eating disorder behaviours’. (P51)
	Facilitates social eating	‘For the first time since developing my ED, I am able to go out and eat at restaurants without fear and anxiety. I eat out and socialise more as a result, which has been freeing’. (P978) ‘For me, calories on menus enable me to eat at restaurants with friends, family and colleagues with minimal anxiety and lead some semblance of a normal life’. (P720)
Emotional distress	Anxiety	‘Makes me extremely uncomfortable and anxious. Makes me want to leave the restaurant’. (P990) ‘I get panic attacks because it reminds me how stressful eating is for me’. (P721)
	Feelings of guilt	‘…When I see the calories next to the items I want to order, my eating disorder immediately makes me feel guilty for wanting something if it is higher on calories than something else that I may not have wanted to order. It makes me feel bad and like a failure if I eat the dish with the higher calories (that I wanted more) compared with another dish with lower calories…’ (P255)
	Judgement from others	‘The impact it has had is it makes me feel more nervous going out with people. I worry if I pick something off the menu that is high in calories, people will judge me’. (P619)
	Negative impacts on social experiences	‘I definitely now feel less comfortable to eat out and can’t help but see the numbers. It has contributed to me avoiding going out for meals socially’. (P16) ‘It stops me enjoying what should be a special occasion, e.g. eating with friends and family’. (P914)
Criticism	Limitations of calorie information	‘Calorie count does not equal health value. So it can be misleading’. (P484)
	Frustration	‘I feel that putting this irrelevant calorie information on menus only serves as a negative. For a person without an eating disorder, they may notice the numbers but, on the whole, it doesn’t change their choice in food option. However, for those with an eating disorder, it is extremely distressing and makes an already stressful experience of dining out even more difficult to manage’. (P660) ‘I don't feel they're useful. They only serve to trigger people with eating disorders and annoy those who don't suffer’. (P164)
	Balancing population needs with those of people with eating disorders	‘While I understand that having calories on menus is useful for monitoring health, it can be awful for people with EDs and issues with eating and body image. I feel as though there is a more nuanced way that this information can be presented’. (P779)
Impact on eating disorder recovery	Triggering	‘I will deliberately avoid places that will trigger dangerous anorexic behaviours. Thanks to this new initiative, this is now every establishment with more than 250 employees. I will go through my day starving myself as opposed to entering such an outlet’. (P32) ‘It’s too risky for me to come across that information. It’s a huge trigger when I'm so early in the recovery journey’. (P552)
	Facilitates the eating disorder	‘It makes me instantly decide that if I eat anything on that menu I might as well binge, because those calories aren't acceptable to my brain’. (P34) ‘When I was going through my eating disorder recovery, it made it feel incredibly critical of any food items I otherwise may have tried’. (P63)
	Fear of relapse	‘I find the calorie information distressing. I have to avoid these restaurants/venues. They lead me into negative thought patterns about food. I am recovered from anorexia, but this was a challenging process and seeing the calorie information feels like walking close to the cliff edge’. (P120)
	Challenge to recovery	‘Seeing calories on menus has previously put me back into disordered eating and restricting’. (P156) ‘I suffered a relapse recently, and it was because I slipped back into counting and restricting calories after a meal out in a restaurant. It can be reassuring for my ED to see the numbers but not for me. It feels dangerous and like it is promoting eating disorders’ (P454)
	Ambivalence	‘When I was initially in recovery for anorexia nervosa and did not want to get better, I liked having calories on menus as a means of control and that was what enabled me to start eating out again as I would only go to restaurants and cafes where the calories of food and drinks were on the menu. Then, when I actually wanted to recover, I found it frustrating because I felt like I had to order the lowest calorie thing on the menu and not what I actually wanted. Then I started just going to places without the calories on the menu. Now I am unsure what I think; it depends on the day, week and month’. (P1031)

### Alternative policy options

**Figure 2:**
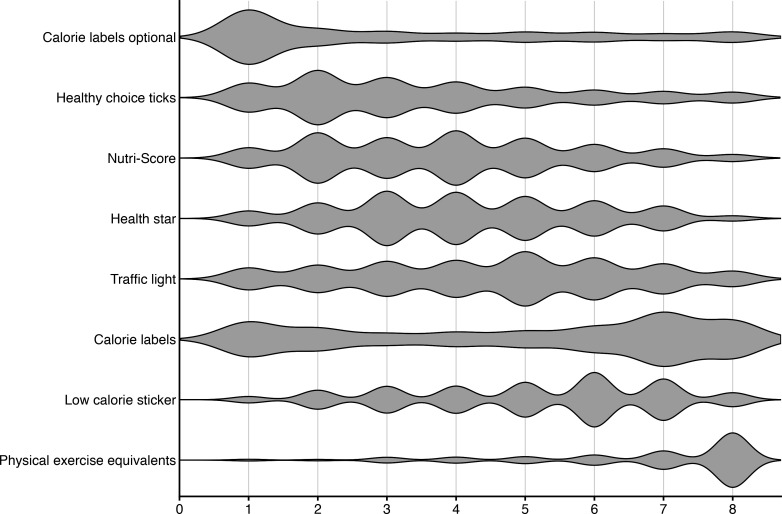
Violin plot of different policy options, in order of preference, with the most preferred option at the top, least preferred at the bottom.

Results from the ranking analyses showed that optional calorie labels were the most popular policy (M=3.08, SD=2.42), with 63.6% ranking the policy in their top three options (see figure 2). In contrast, physical exercise equivalents were consistently rated the lowest (M=6.74, SD=1.77), with 77.6% ranking the policy in their bottom three options. The most controversial policy was the current calorie label policy (M=5.00, SD=2.56), which 31.4% ranked in their top three options and 52.8% ranked in their bottom three options. See online supplemental table S4 for further details. Qualitative responses echoed these findings and highlighted a clear preference for policies that prioritise agency and reduce potential harm (online supplemental table S5). Optional calorie labels were consistently viewed as supporting agency and reducing distress. In comparison, physical exercise equivalents were viewed as distressing and stigmatising, with people expressing concern that they could reinforce disordered beliefs around needing to compensate for eating.

## Discussion

The current study examined the different impact profiles regarding England’s calorie label policy among individuals with disordered eating and explored potential alternative nutritional label policies for the out-of-home sector. Findings showed heterogeneous perceived impacts, with about half reporting negative, a quarter neutral and a quarter positive impacts. Levels of acceptability were similar to those previously reported among individuals with lived experience of an eating disorder living in England.^9^ Advancing this line of research, the latent class analyses pointed towards further nuance in the impacts of the calorie labelling policy on individuals with lived experience of disordered eating. Notably, these were captured as five distinct impact groups: a positive impact group, a neutral impact group and three negative impact groups. The three negative groups differed both on overall levels of acceptance and perceived impact, whereby a moderately negative group emerged, and avoidance of venues with calorie labels distinguished between a highly negative group with avoidance and a highly negative group without avoidance.

This differentiation among not only negative, neutral and positive but also different types of negative impacts has important practical implications for clinical practice, as negative impacts with avoidance were more likely to be reported by individuals in more acute stages of their eating disorder compared with those further along with their recovery. Therefore, gradual exposure to calorie labels and strategies for dealing with distress may form important steps for eating disorder treatment.

Findings underscore that calorie labels impact not only those with a current disorder but also those with a history of an eating disorder. This was further reflected in the qualitative results, whereby participants reported difficulties with calorie labels posed for their recovery and a fear of relapse. However, others reported finding calorie labels helpful for recovery and enabling them to eat out socially, something they were previously unable to do due to their eating disorder. Eating disorders are highly recurrent in nature, with approximately 30%–40% of patients relapsing within 10 years of successful treatment.^29 30^ Public health policies, therefore, need to consider not only the needs of people with current eating disorders but also those with past histories to reduce the risk of relapse and aid recovery.

In line with our hypothesis, people who engaged in binge eating were less likely to be in a negative impact group and were more likely to be in either the neutral or positive impact groups. No other eating disorder behaviours differed between the groups; however, high weight/shape concerns were linked with a lower likelihood of belonging to the *highly negative without avoidance* group, reflecting the ‘recovered’ nature of individuals. High weight/shape concerns were linked with a higher likelihood of belonging to the *positive* group, potentially reflecting that individuals benefitting from calorie labels may attribute ‘positive impacts’ to their ability to control their calorie intake to impact their weight/shape. Hence, positive impacts may refer to both adaptive impacts (ie, better relationships with food, more regulated eating and improved social experiences) and maladaptive impacts (ie, increased calorie counting and more rigid eating). Relatedly, our qualitative theme of ‘reassurance’ could hypothetically reflect a position that is influenced by symptoms such as active dietary restriction. However, our quantitative findings suggest that around a quarter of participants report positive impacts of the policy and that these participants can be characterised by different demographic and symptom profiles, such as binge eating, higher BMI and lack of past eating disorder treatment. Thus, dietary restriction is unlikely to be a key factor driving experiences of reassurance. However, our lived experience consultation suggests that, for people with long-standing restrictive eating disorders, out-of-home calorie labels can improve quality of life by facilitating social eating experiences that would not otherwise be possible. Consequently, our qualitative themes should be interpreted as reflecting views across a range of presentations. Further in-depth qualitative research is needed to further explore the complex relationship between policy impacts and variables such as age, gender, ethnicity, recovery stage and symptom profile. However, as our study was based on an anonymous online survey, it is not possible to distinguish these impacts, and further research should investigate the nuances of impacts using more in-depth qualitative designs.

While both positive and negative impacts of calorie labels on people with lived experience of eating disorders have previously been noted,^4^ this was the first study to investigate whether other nutritional labelling options may be perceived as more beneficial in efforts to mitigate reported harms. Findings showed that optional calorie labels, whereby people can access additional calorie information through a QR code or similar method, were the most popular choice. Participants noted that this policy would reduce the negative impact on people who find the labels harmful, while also providing calorie information to those who find it helpful. Another popular policy was ‘healthy choice ticks’, whereby menu items are highlighted if they meet certain nutritional standards. Participants noted that such a policy provides a more holistic approach to nutrition that goes beyond caloric information while also reducing potential harms commonly associated with calorie labels (eg, calorie counting). In contrast, participants consistently rated physical exercise equivalents as their least preferred option, indicating that the policy would promote harmful ideas around being ‘entitled’ to eat certain foods only if one has done sufficient exercise.

The current study had several strengths, including the broad range of participant characteristics not previously captured in other studies. However, some limitations should be noted. First, the study relied on a self-selecting sample that may have been particularly motivated to share their experiences. While we tried to mitigate this issue through utilising several recruitment sources, including a paid participant pool, more extreme views might be more likely to be captured in the current sample. Second, our study focused solely on perceived impacts and was not able to capture real-world behaviour. Third, all participants had experience with calorie labels as a public policy, which may have impacted their scoring of the policy in the ranking analysis compared with other policies which they may not have encountered previously. Lastly, the current study did not include views from young people under the age of 16. Our findings showed that age was a significant factor distinguishing impact groups, with young people least likely to be captured in the positive impact group. Adolescents are both at the highest risk of developing disordered eating^31^ and are among the highest consumers of out-of-home foods,^32^ placing them at particular risk for negative consequences. However, little is known about their experiences with calorie labels and future research focusing on young people more specifically is needed. Additionally, further research should explore potential mechanisms and moderating factors that can explain why certain anti-obesity policies have negative impacts on individuals with eating disorders.

Our analysis highlights the heterogeneous impacts of England’s calorie labelling policy on individuals with lived experience of disordered eating, with many reporting negative impacts but also many individuals reporting positive impacts, highlighting helpful aspects of aiding recovery and facilitating social eating. Overall, there was a broad desire for policies that provide personal choice over whether nutritional information is presented, allowing greater agency while reducing distress. These findings provide important insights for both evaluating the effectiveness and implementation of the policy in England and international contexts, considering similar nutritional labelling policies.

## Supplementary material

10.1136/bmjph-2025-003666 online supplemental file 1

## Data Availability

Data are available upon reasonable request.
