# Supplementary material for: Views of people with disordered eating on current and alternative out-of-home calorie labelling policies in England: a mixed-methods survey
Source: BMJ Public Health. 2026 Apr 15;4(2):e003666. doi: 10.1136/bmjph-2025-003666 (PMC13084807; doi:10.1136/bmjph-2025-003666)

Supplementary information

Table S1: Operationalisation of DSM-5 Eating Disorder Diagnoses

Diagnosis	Study Criteria
Probable Anorexia Nervosa (AN)	Current BMI percentile < 10; AND compensatory behaviors (self-induced vomiting, laxative misuse, fasting, or driven exercise) OR fear of weight gain or becoming fat over the past 3 months; AND extreme weight or shape concerns over the past 3 months
Probable Bulimia Nervosa (BN)	Binge eating (defined as having unusually large amounts of food with a sense of loss of control) occurs at least once per week over the past 3 months; AND compensatory behaviors (self-induced vomiting, laxative misuse, fasting, or driven exercise) also occur at least once per week over the past 3 months; AND extreme weight or shape concerns over the past 3 months; AND not meeting criteria for AN
Probable Binge Eating Disorder<br>(BED)	Binge eating (defined as having unusually large amounts of food with a sense of loss of control) occurs at least once per week over the past 3 months; AND at least three binge-associated features are endorsed (eating rapidly, until uncomfortably full, when not hungry, alone, or feel distressed after overeating); AND marked distress about binge eating is present; AND absence of any compensatory behaviors (self-induced vomiting, laxative misuse, fasting, or driven exercise); AND not meeting criteria for AN or BN
Probable OSFED – Binge/Purge Type	Binge eating (defined as having unusually large amounts of food with a sense of loss of control) occurs either less than once per week OR at full-threshold frequency over the past 3 months; AND compensatory behaviors (self-induced vomiting, laxative misuse, fasting, or driven exercise), either at subthreshold OR at full-threshold frequency; AND extreme weight or shape concerns over the past 3 months; AND not meeting criteria for AN or BN or BED
Probable OSFED - Binge Eating Disorder	Binge eating (defined as having unusually large amounts of food with a sense of loss of control) occurs less than once per week over the past 3 months; AND at least three binge-associated features are

	endorsed (eating rapidly, until uncomfortably full, when not hungry, alone, or feel distressed after overeating); AND marked distress about binge eating is present; AND absence of any compensatory behaviors (self-induced vomiting, laxative misuse, fasting, or driven exercise); AND not meeting criteria for AN or BN or BED or OSFED binge/purge type
Probable OSFED - Restrictive Type	Fear of weight gain or becoming fat OR compensatory behaviors (self-induced vomiting, laxative misuse, fasting, or driven exercise) over the past 3 months; AND extreme weight or shape concerns over the past 3 months; AND not meeting criteria for AN or BN or BED or OSFED binge/purge type or OSFED BED

Table S2. Model fit indices for LCA models.

Model	BIC	AIC	ll	Entropy
2-classes	8604.2	8555.1	-4267.6	.84
3-classes	8138.6	8069.9	-4020.9	.87
4-classes	8046.7	7958.4	-3961.2	.83
**5-classes**	**7923.1**	**7815.1**	**-3885.6**	**.91**

Table S3. Demographics by latent group

	**Moderately negative**	**Highly negative with avoidance**	**Neutral**	**Highly negative without avoidance**	**Positive**	**Total**
Gender						
Man/trans man	15 8.20%	NR	23 9.58%	NR	36 13.74%	82 8.21%
Woman/trans woman	159 86.89%	180 89.11%	211 87.92%	105 93.75%	218 83.21%	873 87.39%
Non-binary	NR	NR	NR	NR	NR	44 4.40%
Ethnicity						
White	156 85.25%	192 95.52%	189 78.75%	99 88.39%	199 76.83%	835 83.92%
Minority ethnicity	27 14.75%	NR	51 21.25%	NR	60 23.17%	160 16.08%
Age in years	30.12 (11.43)	30.56 (9.65)	31.95 (12.85)	30.86 (10.18)	34.92 (13.98)	31.99 (12.17)
Education						
Below degree level	62 34.07%	55 27.50%	96 40.00%	28 25.00%	78 30.12%	319 32.12%
Degree level	120 65.93%	145 72.50%	144 60.00%	84 75.00%	181 69.88%	674 67.88%
Occupation						
NEET	NR	15 8.02%	16 7.08%	NR	26 11.11%	79 8.54%
Employed full-time or part-time	109 64.50%	140 74.87%	152 67.26%	83 76.15%	160 68.38%	644 69.62%
In education	46 27.22%	32 17.11%	58 25.66%	18 16.51%	48 20.51%	202 21.84%
Weight status						
Underweight	31 16.94%	37 18.32%	37 15.35%	12 10.71%	29 11.03%	146 14.59%
Normal weight	69 37.70%	88 43.56%	96 39.83%	64 57.14%	106 40.30%	423 42.26%
Overweight	25 13.66%	NR	30 12.45%	NR	38 14.45%	107 10.69%
Obese	58 31.69%	68 33.66%	78 32.37%	31 27.68%	90 34.22%	325 32.47%

BMI	24.76<br>(7.99)	22.84<br>(8.51)	25.70<br>(8.60)	24.48<br>(9.02)	27.69<br>(10.24)	25.45<br>(9.16)
Recovery status						
Fully recovered	30<br>16.76%	29<br>14.65%	46<br>20.26%	32<br>29.91%	41<br>16.14%	178<br>18.45%
Partially recovered	110<br>61.45%	126<br>63.64%	136<br>59.91%	65<br>60.75%	152<br>59.84%	589<br>61.04%
Not recovered	39<br>21.79%	43<br>21.72%	45<br>19.82%	10<br>9.35%	61<br>24.02%	198<br>20.52%
CIA social	6.07<br>(4.46)	7.64<br>(4.59)	6.76<br>(4.52)	5.20<br>(4.63)	7.16<br>(4.55)	6.74<br>(4.60)
CIA personal	11.68<br>(5.45)	12.72<br>(4.89)	12.27<br>(4.84)	10.29<br>(5.81)	12.95<br>(4.82)	12.21<br>(5.13)
ED Type						
No eating disorder / low risk	39<br>21.31%	38<br>18.81%	34<br>14.11%	30<br>26.79%	25<br>9.51%	166<br>16.58%
Anorexia nervosa	23<br>12.57%	33<br>16.34%	30<br>12.45%	12<br>10.71%	21<br>7.98%	119<br>11.89%
Bulimia nervosa	23<br>12.57%	NR	27<br>11.20%	NR	37<br>14.07%	104<br>10.39%
Binge eating disorder	13<br>7.10%	NR	17<br>7.05%	NR	30<br>11.41%	74<br>7.39%
OSFED - Beating/purging type	45<br>24.59%	38<br>18.81%	72<br>29.88%	21<br>18.75%	83<br>31.56%	259<br>25.87%
OSFED - Low frequency binge eating disorder	NR	NR	NR	NR	NR	13<br>1.30%
OSFED Restricting type	38<br>20.77%	73<br>36.14%	58<br>24.07%	34<br>30.36%	63<br>23.95%	266<br>26.57%
Purging frequency						
Not at all	138<br>75.41%	144<br>71.29%	177<br>73.44%	89<br>79.46%	185<br>70.34%	733<br>73.23%

1-2 times per week	21<br>11.48%	23<br>11.39%	28<br>11.62%	12<br>10.71%	37<br>14.07%	121<br>12.09%
More than twice a week	24<br>13.11%	35<br>17.33%	36<br>14.94%	11<br>9.82%	41<br>15.59%	147<br>14.69%
Binge eating frequency						
Not at all	78<br>42.62%	119<br>58.91%	73<br>30.29%	67<br>59.82%	82<br>31.18%	419<br>41.86%
1-2 times per week	63<br>34.43%	47<br>23.27%	93<br>38.59%	27<br>24.11%	89<br>33.84%	319<br>31.87%
More than twice a week	42<br>22.95%	36<br>17.82%	75<br>31.12%	18<br>16.07%	92<br>34.98%	263<br>26.27%
Excessive exercise frequency						
Not at all	112<br>61.54%	105<br>51.98%	145<br>60.17%	71<br>63.39%	151<br>57.41%	584<br>58.40%
1-2 times per week	33<br>18.13%	40<br>19.80%	47<br>19.50%	13<br>11.61%	50<br>19.01%	183<br>18.30%
More than twice a week	37<br>20.33%	57<br>28.22%	49<br>20.33%	28<br>25.00%	62<br>23.57%	233<br>23.30%
Fasting frequency						
Not at all	93<br>51.10%	85<br>42.29%	102<br>42.32%	64<br>57.14%	106<br>40.30%	450<br>45.05%
1-2 times per week	37<br>20.33%	40<br>19.90%	57<br>23.65%	17<br>15.18%	64<br>24.33%	215<br>21.52%
More than twice a week	52<br>28.57%	76<br>37.81%	82<br>34.02%	31<br>27.68%	93<br>35.36%	334<br>33.43%
Weight/shape concerns	5.01<br>(1.49)	4.99<br>(1.46)	5.12<br>(1.24)	4.19<br>(2.02)	5.35<br>(1.22)	5.03<br>(1.47)
Current comorbid disorder						
No	58<br>31.69%	64<br>31.68%	77<br>31.95%	54<br>48.21%	95<br>36.12%	348<br>34.77%
Yes	125	138	164	58	168	653

	68.31%	68.32%	68.05%	51.79%	63.88%	65.23%
Past comorbid disorder						
No	94	105	135	68	149	551
	51.37%	51.98%	56.02%	60.71%	56.65%	55.04%
Yes	89	97	106	44	114	450
	48.63%	48.02%	43.98%	39.29%	43.35%	44.96%
Neurodiversity						
Yes	78	111	107	45	132	473
	43.33%	55.50%	46.32%	41.67%	51.56%	48.51%
No	102	89	124	63	124	502
	56.67%	44.50%	53.68%	58.33%	48.44%	51.49%

NR = Not reported due to low cell count.

Table S4. Mean ranks, standard deviations, and median ranks for each labelling policy option.

Policy option	M	SD	Median	% in top three	% in bottom three
Calorie labels	5.00	2.56	6	31.4	52.8
Optional calorie labels	3.08	2.42	2	63.6	20.6
Traffic light labels	4.51	1.97	5	31.4	32.6
Physical exercise equivalents	6.74	1.77	8	8.2	77.6
Nutri-Score A-E labels	3.90	1.82	4	42.3	20.0
Health star ratings	4.19	1.75	4	37.5	24.5
Low calorie stickers	5.07	1.83	5	22.8	48.0
Healthy choice ticks	3.51	1.98	3	56.5	17.6

Table S5. Illustrative participant quotes explaining preferences in policy ranking responses.

Policy options	Illustrative Quotes
Calorie labels	“Calorie labels are good so that people know what they are eating and can make sensible choices” (P419)
	“Calorie labels are lower down as they again are a impediment to people with disordered eating and in the throes of Anorexia, I would not for example, have touched anything over 200 calories.” (P531)
Calorie labels as optional (e.g., use QR to get calorie labels)	“Having the calorie or nutritional content of any kind as optional to access through a QR code means I have the choice whether I want to see this information rather than being unavoidably exposed to it” (P277)
	“Putting the QR code labels for calorie choices still allows everyone access to the nutritional value but it is a choice on whether you get to see the nutritional values rather then being forced to see then which, in my instance can be damaging for my mental health” (P823)
Traffic light labels	“Traffic light labels look at overall nutrition content not just calories, this gives me a better indication of whether a meal is balanced rather than how many calories it contains.” (P339)
	“I also am hesitant about traffic lights, as I think they can create the idea that a food is always 'bad', and this can lead to feelings of guilt, shame etc.” (P248)
Physical exercise equivalents	“Physical exercise equivalents are a terrible idea because they reinforce the idea that food needs to be earned or exercise is the punishment for eating” (P258)
	“The physical exercise one would be the least preferred. It would make you feel guilty if you didn't then do the exercise. Also, not everyone can do the same suggested physical exercises” (P553)
Nutri-Score labels A-E	“Nutrition labels A-E I don’t think are widely understood so not very helpful.” (P40)
	“When I lived in the Netherlands I found the A-E system to be helpful as I could still enjoy the foods I wanted without worrying about numbers” (P612)
Health star rating	“I don't particularly like health star ratings and ratings similar because they are quite misleading and don't consider the nutrient density of a food.” (P586)

	"Health star ratings are better because it's a less obsessive way to see what's good and bad for you" (P541)
Low calorie stickers	"Having the low calorie stickers makes it easier to locate the best choice for me without taking too much time to deliberate, stress or draw notice to myself." (P523)
	"Low calorie sticks is a more discrete way of displaying a reduced calorie option without displaying numerical value." (P834)
Healthy choice ticks	"I think the healthy choice ticks are better because whilst they may also be low calorie , it makes it less blazingly obvious to those in recovery and makes it feel like less of an obligation to choose than having a low calorie stickers" (P505)
	"Healthy choice ticks would help me make better decisions, and give a clearer indication of what types of food are healthier choices" (P748)

Figure S1. Flowchart of recruitment and data cleaning process

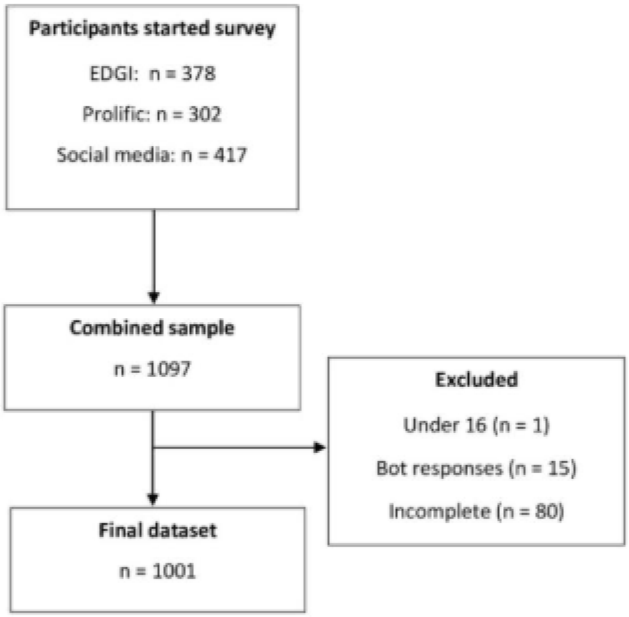

Figure S2. Acceptability and perceived impacts of the calorie label policy.

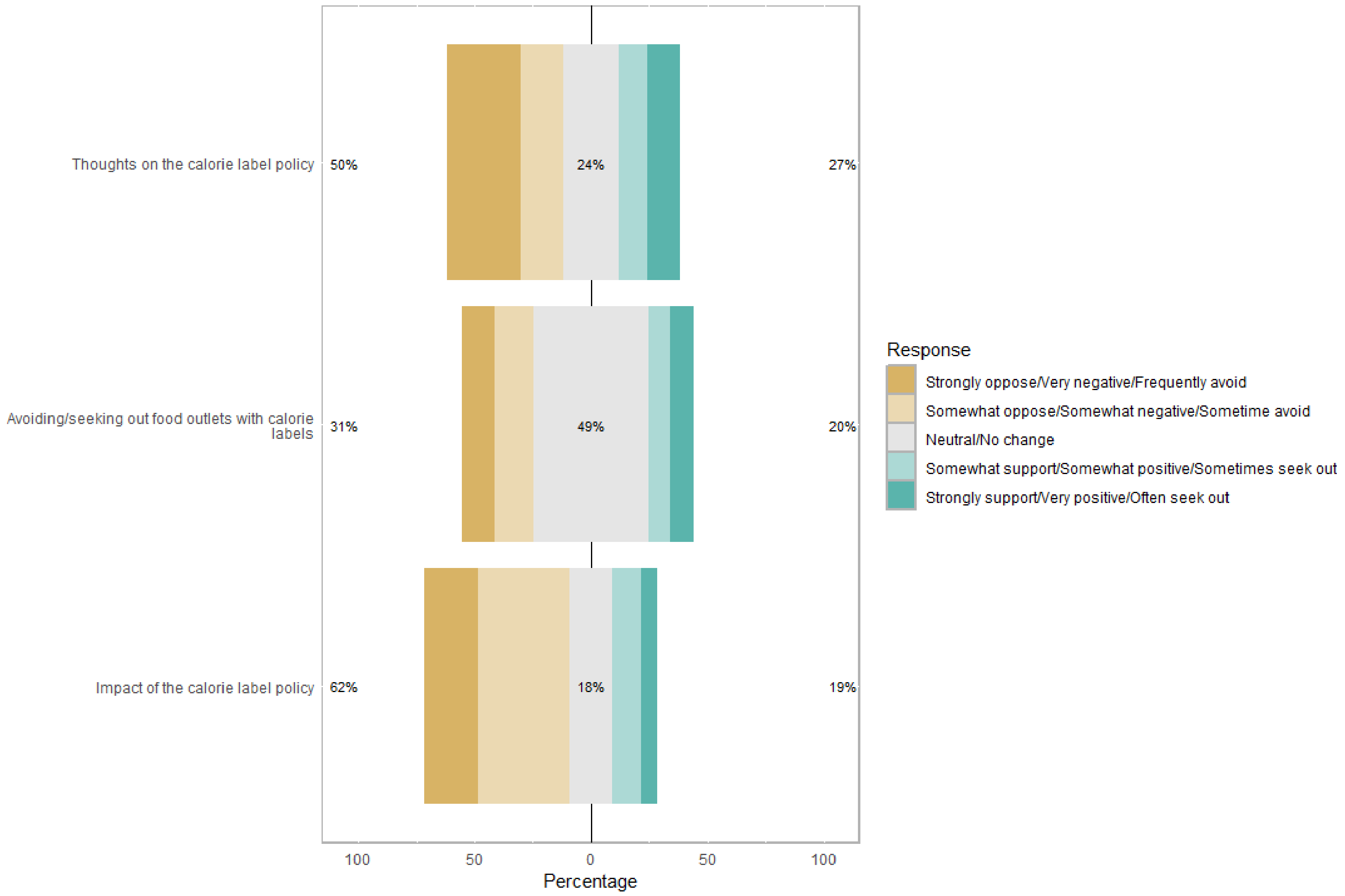

Supplement: online supplemental file 1 [file bmjph-4-2-s001.pdf]
